# A new Miocene baleen whale from Peru deciphers the dawn of cetotheriids

**DOI:** 10.1098/rsos.170560

**Published:** 2017-09-13

**Authors:** Felix G. Marx, Olivier Lambert, Christian de Muizon

**Affiliations:** 1 School of Biological Sciences, Monash University, 25 Rainforest Walk, Clayton, Victoria 3800, Australia; 2 Geosciences, Museum Victoria, Melbourne, Australia; 3 Directorate of Earth and History of Life, Royal Belgian Institute of Natural Sciences, Brussels, Belgium; 4 CR2P (MNHN, CNRS, UPMC, Sorbonne-Université), Département Origines et Évolution, Muséum national d'Histoire naturelle, Paris 75005, France

**Keywords:** Mysticeti, baleen whale, pisco formation, cetotheriidae, evolution, phylogeny

## Abstract

Cetotheriidae are an iconic, nearly extinct family of baleen whales (Mysticeti) with a highly distinct cranial morphology. Their origins remain a mystery, with even the most archaic species showing a variety of characteristic features. Here, we describe a new species of archaic cetotheriid, *Tiucetus rosae*, from the Miocene of Peru. The new material represents the first mysticete from the poorly explored lowest portion of the highly fossiliferous Pisco Formation (allomember P0), and appears to form part of a more archaic assemblage than observed at the well-known localities of Cerro Colorado, Cerro los Quesos, Sud-Sacaco and Aguada de Lomas. *Tiucetus* resembles basal plicogulans (crown Mysticeti excluding right whales), such as *Diorocetus* and *Parietobalaena*, but shares with cetotheriids a distinct morphology of the auditory region, including the presence of an enlarged paroccipital concavity. The distinctive morphology of *Tiucetus* firmly places Cetotheriidae in the context of the poorly understood ‘cetotheres’ *sensu lato*, and helps to resolve basal relationships within crown Mysticeti.

## 1. Introduction

Cetotheriidae are a group of relatively small and mostly extinct baleen whales sharing a highly distinct cranial morphology [1–3]. The existence and, for the most part, scope of this family are widely agreed on [1,2,4–7], but this consensus is a relatively recent phenomenon. The term ‘cetothere’ has a long history as a wastebasket taxon covering any extinct chaeomysticete (toothless baleen whale) that does not unambiguously fall within one of the living families. A detailed re-examination of this group led to a revision dividing ‘cetotheres’ into Cetotheriidae *sensu stricto*, the family as understood today [1–3]; and ‘cetotheres’ *sensu lato*, a para- or polyphyletic assemblage of species whose phylogenetic relationships are highly uncertain [1,2,4,6,8–11].

Cetotheriids had their heyday during the Late Miocene, when they rivalled rorquals in diversity and enjoyed a global distribution [4,5]. During the Pliocene, cetotheriids declined, with herpetocetines and neobalaenines (*sensu* [12]) remaining as the only—albeit initially abundant—lineages [3,6,13,14]. During the Pleistocene, herpetocetines also disappeared [13], leaving the pygmy right whale *Caperea marginata* as the sole modern survivor [12,15]. Phylogenetic analyses generally agree that cetotheriids are related to balaenopteroids [2,5,15], but the time, place and morphological context of their origin remain obscure: like most of the major baleen whale lineages, cetotheriids suffer from a dearth of transitional fossils that could illuminate their place within crown Mysticeti and, in particular, their relationship(s) with ‘cetotheres’ *sensu lato*. Here, we report just such a transitional fossil, in the form of a new, well-preserved Miocene mysticete from the highly fossiliferous Pisco Formation of Peru (e.g. [16–18]). Our new specimen combines an overall archaic, ‘cetothere’ *sensu lato*-like morphology with features typical of cetotheriids, and thus helps to place the latter in the context of basal mysticete phylogeny.

## 2. Material and methods

The material described here was collected by one of us (C.M.) in 1987, and prepared using 5% formic acid. Morphological terminology follows Mead & Fordyce [19], unless indicated. For the figures, photographs of the specimen were digitally stacked in Photoshop CS6. To determine evolutionary affinities, we added the new material to the total evidence data matrix of [15]. In addition, we altered the latter slightly by adjusting Character 154, ‘Articulation of anterior process of periotic and tympanic bulla’, to include a new state, ‘1: accessory ossicle fused to periotic but still clearly defined anteriorly’. This change was made to reflect the presence of a previously unscored transformation series, leading from an unfused to an entirely fused and indistinct accessory ossicle of the tympanic bulla [20]. The resulting 3-state character was ordered. Scoring changes arising from this amendment only affect the oldest putative crown mysticetes included in this analysis (*Mauicetus parki* and ZMT 67), which have previously been suggested to cluster with at least one ‘cetothere’ *sensu lato, Aglaocetus moreni* [4]. The cladistic analysis was run in MrBayes 3.2.6, using the same settings as in [15], on the Cyberinfrastructure for Phylogenetic Research (CIPRES) Science Gateway [21].

### 2.1. Institutional abbreviations

HMN, Hiwa Museum of Natural History, Hiwa, Japan; IRSNB, Institut Royal des Sciences Naturelles de Belgique, Brussels, Belgium; MAB, Oertijdmuseum ‘The Groene Poort’, Boxtel, the Netherlands; MFM, Mizunami Fossil Museum, Gifu, Japan; MNHN, Muséum national d'Histoire naturelle, Paris, France (palaeontological collection, MNHN.F.); NMR, Natuurhistorisch Museum Rotterdam, the Netherlands; NMRA, National Museum of the Republic of Adygeya, Maikop, Russia; NMNZ, Museum of New Zealand Te Papa Tongarewa, Wellington, New Zealand; OM, Otago Museum, Dunedin, New Zealand; OMNH, Osaka Museum of Natural History, Osaka, Japan; OU, University of Otago Geology Museum, Dunedin, New Zealand; SMNH, Saitama Museum of Natural History, Saitama, Japan; UCMP, University of California Museum of Paleontology, Berkeley, USA; USNM, National Museum of Natural History, Smithsonian Institution, Washington, DC, USA; ZMA, Naturalis, Leiden, The Netherlands, referring to material formerly housed at the Zoologisch Museum, Amsterdam, The Netherlands; ZMT, Fossil mammals catalogue, Canterbury Museum, Christchurch, New Zealand.

## 3. Systematic palaeontology

Cetacea Brisson, 1762

Neoceti Fordyce and Muizon, 2001

Mysticeti Gray, 1864

Chaeomysticeti Mitchell, 1989

Cetotheriidae Brandt, 1872; *sensu* Fordyce and Marx, 2013

*Tiucetus* gen. nov.

**LSID.** urn:lsid:zoobank.org:act:47EF3345-A286-4FA6-AC6C-DEC9C4BFD9C5

**Type species.** *Tiucetus rosae* gen. et sp. nov.

**Etymology.** From Quechua *tiu*, meaning sand, with reference to the coastal deserts of Peru.

**Diagnosis.** As for the type and only species.

*Tiucetus rosae*, sp. nov.

Figures 2–9

**LSID.** urn:lsid:zoobank.org:act:BE8A9A89-2ED7-42CA-8CF9-BABB7199F4DD

**Holotype.** MNHN.F. PPI261, a partial cranium preserving most of the braincase, the bases of both supraorbital processes, both periotics and tympanic bullae, both mallei and stapes, and the central portion of the rostrum except for the palatines.

**Figure 1.**
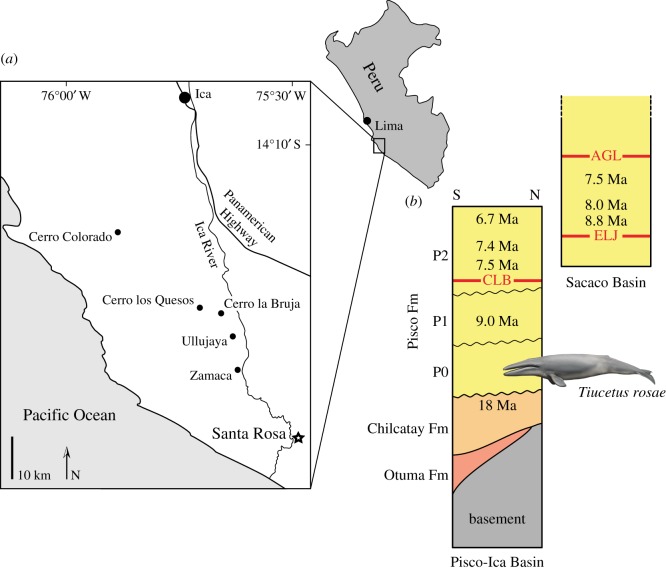
Locality and horizon of the holotype of *Tiucetus rosae.* (*a*) Schematic map of the Pisco-Ica Basin, modified from [22], showing the position of the Santa Rosa (black star) and other fossil-bearing localities of the Pisco and Chilcatay formations. (*b*) Succession of lithological units along a section at Cerro Las Tres Piramides, a locality in the Pisco-Ica Basin approximately 25 km northwest of Santa Rosa (modified from [23]). The drawing of a cetotheriid marks the provenance of the holotype of *Tiucetus rosae* from allomember P0. To the right of the section is a partial overview of the Pisco Formation as exposed in the Sacaco Basin (modified from [18,24]), showing the proposed order of the vertebrate horizons of Cerro la Bruja (CLB), El Jahuay (ELJ) and Aguada de Lomas (AGL). Note the mismatch between the proposed ages for CLB and ELJ.

**Locality and horizon.** Santa Rosa, Pisco Basin, Peru; approx. coordinates: 14°47′22.9′′ S, 75°30′22.5′′ W (figure 1). The holotype of *Tiucetus rosae* came from the poorly explored lowest part of the Pisco Formation (allomember P0 [23], which includes the Santa Rosa vertebrate level [25]), and therefore predates all other mysticetes previously described from the Miocene of Peru [1,26–28]. The specimen was associated with several skulls of a small, kentriodontid-like delphinidan, which also occurs at other P0 localities of the Pisco Basin, such as Malpaso (G. Bianucci 2017, personal communication). Other mysticetes from this allomember include larger specimens reminiscent of the similarly aged *Pelocetus*, and at least one species broadly resembling Late Miocene cetotheriids like ‘*Cetotherium*’ *megalophysum* and *Herentalia* ([22]; F.G.M., personal observation). Overall, the P0 assemblage appears considerably more archaic than that found at the well-known Pisco localities of Cerro Colorado, Cerro Los Quesos, Cerro la Bruja and Aguada de Lomas [16,18,25,29].

The strata that yielded the holotype of *Tiucetus rosae* rest unconformably on the latest Oligocene to Early Miocene Chilcatay Formation. Sediment samples from the skull, associated specimens and the locality of Santa Rosa itself failed to yield microfossils, and there is currently no other direct dating evidence available for P0. Nevertheless, dates from the underlying Chilcatay Formation and the overlying P1 allomember of the Pisco Formation constrain the deposition of P0 to between 17.8 and 9.0 Ma [23]. Furthermore, dated ash beds from within the P1 and P2 allomembers suggest that both were deposited over no more than 1–1.5 Myr each [23]. Assuming similar rates of sedimentation for P0 would yield an approximate age of 11–9 Ma (Tortonian) for *Tiucetus*, which is consistent with the occurrence of a ‘typical’ Late Miocene cetotheriid morphotype (i.e. one broadly resembling ‘*Cetotherium*’ *megalophysum* and *Herentalia*) in the same unit.

However, the situation is complicated by an apparent mismatch between faunal and radiometric data from the Pisco-Ica and Sacaco basins. Of particular relevance here is the age of the El Jahuay vertebrate level (ELJ), which is among the oldest vertebrate horizons exposed in the Sacaco Basin, and has been K/Ar-dated to older than 8.8 Ma [18]. Based on its vertebrate fauna, ELJ appears to be stratigraphically above the Cerro La Bruja vertebrate level (CLB) of the Pisco-Ica Basin [18,25,30], yet diatom assemblages and more recent radiometric dates from the localities of Cerro La Bruja and Cerro Los Quesos place CLB between 7.5 and 8.5 Ma [23,31,32]. If CLB has been correctly dated, then a revision of the dating evidence from the Sacaco Basin appears to be in order. Alternatively, if the ELJ estimate is correct, CLB, and indeed all of the Pisco Formation exposed in the Pisco-Ica Basin, may be older than recently suggested [23,31,32]. In this scenario, a conservative estimate for the age of P0 might be late Middle Miocene (Serravallian; 13.8–11.6 Ma), which may be further supported by the markedly archaic aspect of the cetacean assemblage from this unit relative to P1 and P2 (see above).

In the light of these conflicting hypotheses, the age of *Tiucetus rosae* must fall somewhere between 17.8 and 9.0 Ma, and plausibly within either the Serravallian or the early Tortonian period, depending on the resolution of the mismatch between the Pisco-Ica and Sacaco basins. A Serravallian age is supported by the mainly archaic cetacean assemblage of P0 and radiometric dates from the Sacaco Basin, although the more recent dating evidence from the Pisco-Ica Basin does not exclude the possibility of an early Tortonian age. Direct evidence for the age of P0 and a re-examination of the radiometric data from the Sacaco area are required to resolve this question.

**Etymology.** Named after the type locality, Santa Rosa.

**Diagnosis.** Small-sized baleen whale differing from all other chaeomysticetes except cetotheriids in having a distally expanded compound posterior process of the tympanoperiotic (hereafter: posterior process) with a well-defined external surface and a partially floored facial sulcus; further differs from eomysticetids, balaenids, *Diorocetus*, and the cetotheriids *Brandtocetus*, *Cetotherium*, *Herpetocetus* and *Piscobalaena* in having a squamosal cleft; from eomysticetids in having comparatively short nasals, a more anteriorly projected supraoccipital and parietal, and a tympanic bulla that is rotated so that the inner posterior prominence faces dorsally; from balaenids in having a transversely compressed anterior process of the periotic that is underlapped by the lateral lamina of the pterygoid, and in lacking an arched rostrum; from balaenopterids and eschrichtiids in having a supraorbital process that gradually descends from the skull vertex, a broadly triangular ascending process of the maxilla, and a pars cochlearis that is not cranially elongated; from *Isanacetus*, *Parietobalaena* and ‘*Diorocetus*’ *chichibuensis* in having a narrow body of the periotic with no sign of lateral inflation, and a more prominent inner posterior prominence of the tympanic bulla that is not medially and anteriorly retracted; from *Diorocetus* in having the inner posterior prominence of the tympanic bulla developed as a bulbous dorsal projection on the involucrum, and in lacking a ridge posteriorly bordering the facial sulcus on the posterior process; from all cetotheriids and *Titanocetus* in having a broadly triangular ascending process of the maxilla, a well-developed ascending process of the premaxilla extending posteriorly as far as the maxilla, and a nasal that only moderately tapers posteriorly; and from all cetotheriids except *Cephalotropis* and *Joumocetus* in having parietals that are well exposed on the skull vertex.

## 4. Description

### 4.1. Overview

The braincase and vertex are relatively complete and well preserved, except for the left postorbital process of the squamosal and the ventralmost portions of both pterygoids. The outer portions of both supraorbital processes and the rostrum are missing. Measurements of the cranium are presented in table 1.

**Table 1.** Measurements of the cranium and ear bones of *Tiucetus rosae* (in mm) (e, estimate).

*cranium excluding ear bones*	
condylobasal length, as preserved	585
bizygomatic width	401
bicondylar width	114
width of the foramen magnum	44
height of the foramen magnum	47
maximum width across exoccipitals	267
length of nasal (right), as preserved	104
width across ascending processes of premaxillae, posteriorly	21
width across nasals, anteriorly	37 (e)
width across nasals, posteriorly	9
maximum width of the narial fossa	54
width of the intertemporal constriction	117
width of the temporal fossa at the tip of the zygomatic process of the squamosal	120
length of the zygomatic process of the squamosal (right)	105
width of the postglenoid process (right)	91
length of the fossa for sternocephalicus (right)	57
height of the fossa for sternocephalicus (right)	18
diameter of the external acoustic meatus at the medial border of the postglenoid process (right)	21
length of the pterygoid exposure between the palatine and the falciform process of the squamosal	30
maximum diameter of the foramen pseudovale (right)	23
length of the pterygoid sinus fossa, measured from the posterior edge of the falciform process (left)	49
width of the pterygoid sinus fossa at the posterior edge of the falciform process (left)	42
width of the medial lamina of pterygoid at the centre of the pterygoid sinus fossa	19
width of the vomer at the maxillo-palatine suture	57
length of the paroccipital fossa, including the portion on the posterior process of the tympanoperiotic (left)	29
width of the jugular notch (left)	22
maximum width across basioccipital crests	82
minimum distance between basioccipital crests	21
maximum width of the basioccipital crest (left)	29
length of the basioccipital crest (left)	45
*periotic*	
length of the anterior process anterior to the mallear fossa	34.9
length of the pars cochlearis anterior to the fenestra cochleae	25.6+
width of the pars cochlearis medial to the fenestra vestibuli	12
diameter of the fenestra cochleae (left)	4.2
length (long axis) of the posterior process	60.5
maximum anteroposterior diameter of the external surface of the posterior process (left)	31.4
maximum diameter of the lateral end of the facial canal (left)	10.5
*tympanic bulla and malleus*	
length (right)	71.8
width anterior to the sigmoid process (right)	44.2
maximum width of the aperture of the tympanic cavity (left)	16.2
height of the sigmoid process, measured from the base of the sigmoid cleft (right)	29.8
maximum height of the median furrow (right)	19.3
malleus length (anteroposterior)	7.8
malleus width	13.4

### 4.2. Maxilla, premaxilla and nasal

**Figure 2.**
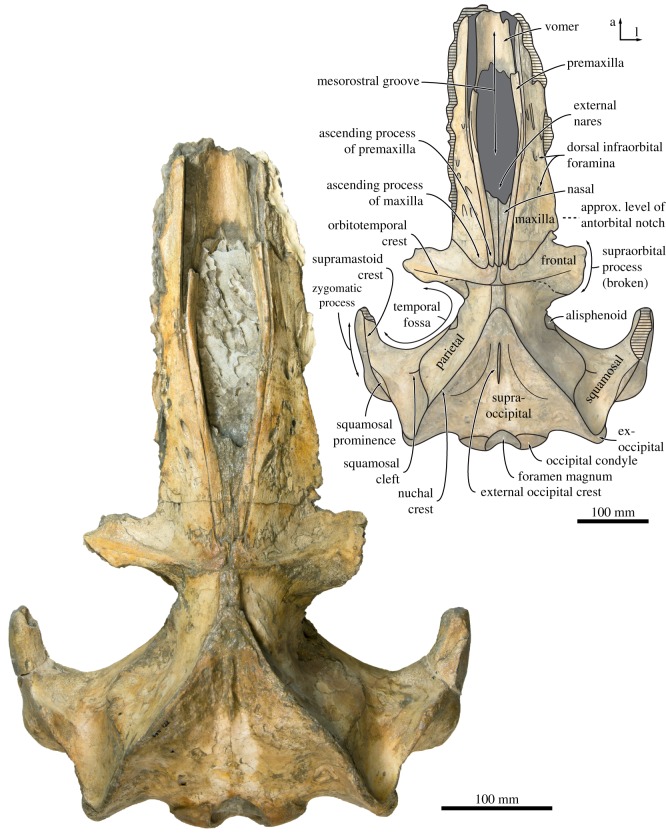
Cranium of *Tiucetus rosae* (MNHN.F. PPI261, holotype), in dorsal view (a, anterior; l, lateral).

**Figure 3.**
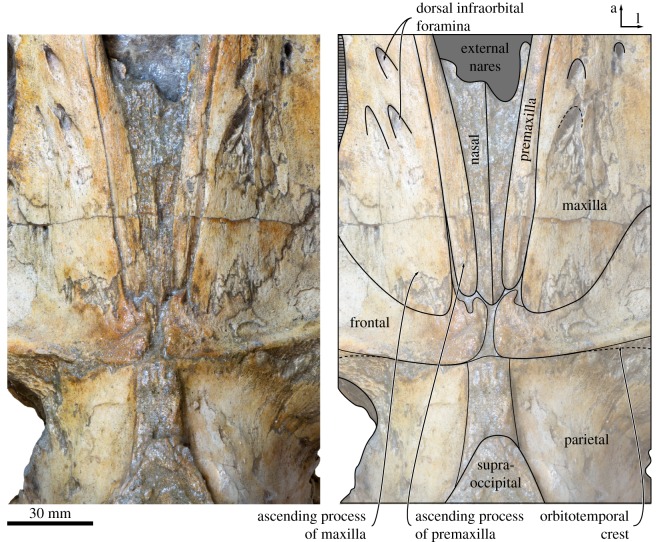
Close-up of the cranial vertex of *Tiucetus rosae* (MNHN.F. PPI261, holotype), in dorsal view (a, anterior; l, lateral).

In dorsal view, the ascending process of the premaxilla is robust and parallel-sided, and extends posteriorly as far as the maxilla (figures 2 and 3). The suture between the premaxilla and the maxilla is open along its entire preserved length, suggesting rostral kinesis. Centrally on the rostrum, the premaxillae define a broad and elongate narial fossa. Anterior to the narial fossa, the premaxillae are lost but, judging from the shape of the adjacent maxilla, appear to have widened anteriorly. The nasal is relatively elongate and extends anteriorly far beyond the inferred level of the antorbital notch. Posteriorly, the nasal tapers, but still makes a robust contact with the frontal and extends to about the same level as the premaxilla and maxilla.

The ascending process of the maxilla is broadly triangular and extends roughly halfway across the length of the frontal (figures 2 and 3). Adjacent to the anterior portion of the nasal, each maxilla bears a cluster of three large dorsal infraorbital foramina with associated, posteromedially oriented sulci. The posteriormost of these foramina opens posteriorly in the direction of the ascending process of the maxilla, and may hence be homologous with the primary dorsal infraorbital foramen of other cetotheriids (*sensu* [7]). Additional foramina, some of comparable size, open further anteriorly on the maxilla and are associated with (mostly anteriorly) oriented sulci.

**Figure 4.**
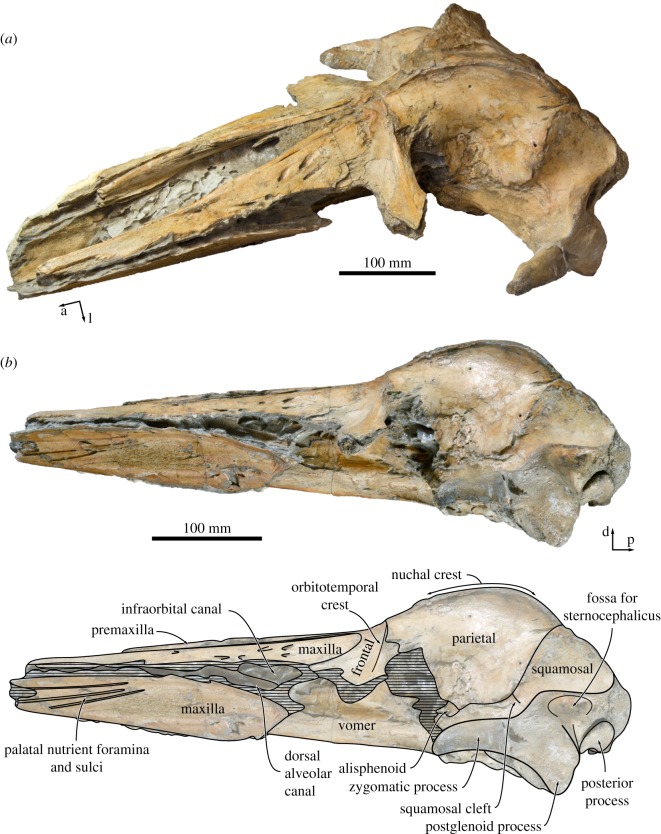
Cranium of *Tiucetus rosae* (MNHN.F. PPI261, holotype) in (*a*) oblique anterodorsal and (*b*) lateral view (a, anterior; d, dorsal; l, lateral; p, posterior).

In lateral view, the dorsal margin of the rostrum is straight, with the premaxilla and nasal being flush with the maxilla (figure 4*a*,*b*). The lateral edge of the maxilla is dorsoventrally flattened. Posteriorly, the medial portion of the maxilla gradually expands in height, with its ventral border gradually descending towards the inferred level of the orbit. Breakage has exposed the infraorbital canal, including what appears to be its bifurcation into the dorsal alveolar canal and the infraorbital canal proper.

In ventral view, the maxilla is transversely concave, with its medial portion steeply descending along the vomer to form a pronounced palatal keel (figures 4*b* and 6). Posteriorly, each maxilla preserves the posteriorly concave, anteromedially directed outline of the maxillo-palatine suture, similar to that of *Parietobalaena palmeri* (USNM 16119). Somewhat anterior to this suture, approximately 20–25 mm from the medial border of the maxilla, a poorly preserved groove probably represents the palatine sulcus. The anterior portion of each maxilla bears several elongate palatal nutrient foramina and sulci. The more lateral of these sulci are oriented anteriorly, whereas the more medial ones point anteroventrally or even anteroventromedially and extend far on to the palatal keel.

### 4.3. Frontal

In dorsal view, the frontal is exposed on the cranial vertex for a length of about 18 mm (figure 3). The interfrontal suture is evident, and there is no narial process. A sharp orbitotemporal crest runs from the vertex along the centre of the supraorbital process, before gradually becoming less distinct and finally disappearing completely roughly halfway between the sagittal plane and the inferred position of the orbit. The dorsal surface of the supraorbital process is smooth, with no obvious foramina. In anterior view, the supraorbital process is straight to slightly concave, and gently slopes ventrally as it descends from the vertex (figure 7*a*). In lateral view, the portion of the frontal exposed at the vertex is oriented at an angle relative to the rostrum, and rises dorsally towards its suture with the parietal. In ventral view, there is a well-developed, sharp preorbital ridge, bordered anteriorly by a wide, slightly concave area that presumably once accommodated the infraorbital plate of the maxilla (figure 6).

### 4.4. Parietal

**Figure 5.**
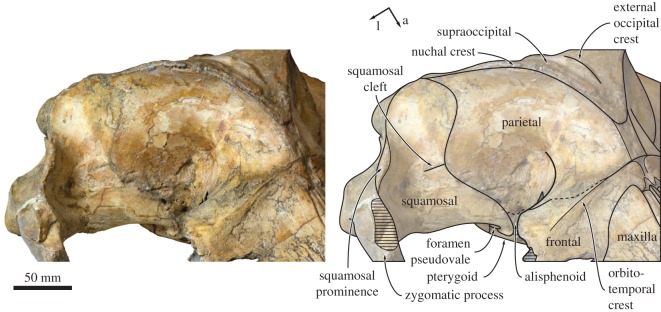
Right temporal fossa of *Tiucetus rosae* (MNHN.F. PPI261, holotype) in anterolateral view (a, anterior; l, lateral).

In dorsal view (figure 3), the parietals are broadly exposed on the vertex and form a broad (18 mm) sagittal ridge that contrasts with the narrow crest of *Parietobalaena* (*P. palmeri*: USNM 10677, 16119; *P. yamaokai*: HMN F00042). Anteriorly, the parietal overrides the posterior portion of the frontal, extending all the way to the orbitotemporal crest and on to the posteromedial corner of the supraorbital process. Where it forms the intertemporal constriction, the parietal is convex transversely, but then becomes partially concave (both anteroposteriorly and dorsoventrally) inside the temporal fossa. In lateral view, the parieto-squamosal suture is sigmoidal and descends almost vertically from the nuchal crest, before turning 90° and terminating at the alisphenoid (figure 5). Along the suture, both the parietal and the adjacent squamosal bulge anterolaterally into the temporal fossa. There is no tubercle where the parieto-squamosal suture meets the nuchal crest, and no postparietal foramen.

### 4.5. Squamosal

The right squamosal is complete, except for some damage to the zygomatic process and some minor erosion of the postglenoid process. In dorsal view, the squamosal, parietal and frontal define a temporal fossa that is wider transversely than it is long (figure 2). The zygomatic process is robust, oriented slightly anterolaterally, and bears a low, rounded supramastoid crest along its posterior portion. The squamosal fossa is elongate, convex anteriorly and concave posteriorly. Inside the fossa, there is a short (approx. 20 mm) squamosal cleft. Anteroventrally, the border of the squamosal is smooth, with no sign of a squamosal crease. Posteriorly, the nuchal crest terminates anterior to the level of the occipital condyles.

In lateral view, the postglenoid process is robust, triangular in outline, and oriented ventrally or slightly posteroventrally, with its posterior border being slightly concave (figure 4*b*). The lateral face of the postglenoid process is flattened and projects beyond the outer surface of the zygomatic process, thus creating a bony eminence where the two processes meet. The zygomatic process is also robust, with approximately parallel dorsal and ventral borders. Dorsal to the base of the postglenoid process, there is a well-developed, bluntly triangular squamosal prominence (figure 5). Immediately posteroventral to the prominence, the squamosal is excavated by a moderately sized, deep fossa for the sternocephalicus, which is separated from a second, smaller and shallower fossa just below by a low, rounded horizontal ridge.

In posterior view, the squamosal projects laterally well beyond the lateral border of the exoccipital (figure 7*b*). Unlike in certain cetotheriids, such as *Metopocetus* (*M. hunteri*, NMR9991-07729) and ‘*Cetotherium*’ *megalophysum* (USNM 205510), the posterior meatal crest does not clearly extend on to the base of the postglenoid process, except for a faint, short (approx. 20 mm) ridge present on the left side only. The postglenoid process is wide and, accounting for breakage, appears to have been broadly parabolic in outline, as seen in a somewhat more elongated form in *Parietobalaena* (e.g. *P. campiniana*, IRSNB M399-R4018; *P. palmeri*, USNM 16229). Ventrally, the postglenoid process extends well below the level of the paroccipital process.

**Figure 6.**
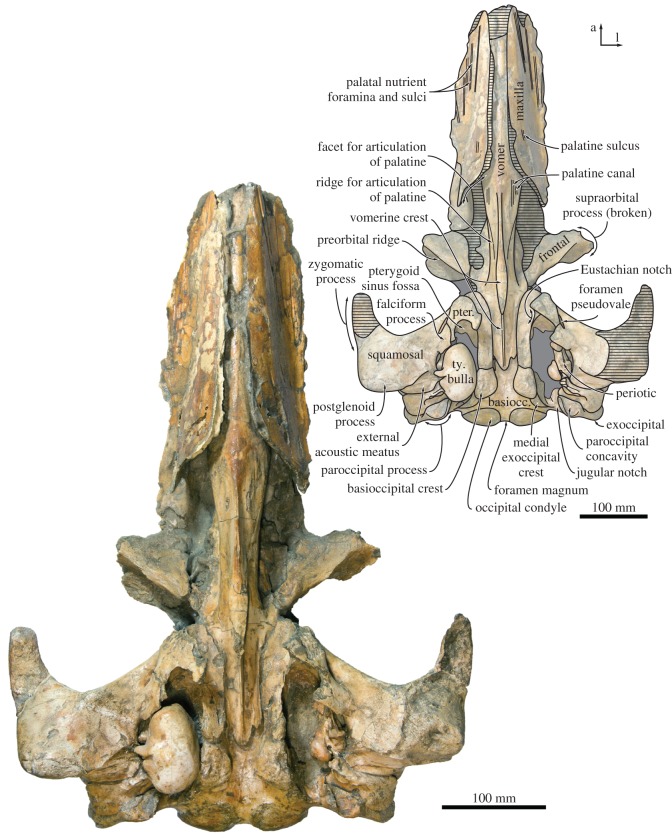
Cranium of *Tiucetus rosae* (MNHN.F. PPI261, holotype), in ventral view (a, anterior; basiocc., basioccipital; l, lateral; pter., pterygoid; ty. bulla, tympanic bulla).

In ventral view, the falciform process is robust. Its posterior border is slightly notched, closely apposed to the anterior border of the tympanic bulla, and underlaps the anterior process of the periotic (figures 6 and 8). The postglenoid process is oriented transversely, with no evidence of twisting as seen in *Piscobalaena* and herpetocetines (figure 6). The anterior surface of the postglenoid process is convex transversely, except for its medialmost portion, which is markedly concave and appears as if pinched. The transition from the convex to the concave portion of the postglenoid process is aligned with a bony protuberance, which is located close to the rim of the temporal fossa. Medially, the postglenoid process directly borders the shallow sigmoid fossa, with no intervening anterior meatal crest. The external acoustic meatus is parallel-sided, with the posterior meatal crest descending only slightly on to the posterior process (figure 8).

### 4.6. Supraoccipital, exoccipital and basioccipital

In dorsal view, the supraoccipital is triangular with a narrow, pointed apex and sigmoidal lateral borders (i.e. nuchal crests). As in *Isanacetus* and *Parietobalaena*, but unlike in other cetotheriids, the tip of the supraoccipital extends anteriorly just beyond the level of the zygomatic process of the squamosal (figure 2). The nuchal crest is oriented dorsally and does not overhang the temporal fossa (figure 5). Posterior to its apex, the supraoccipital shield is initially flattened, but then abruptly turns concave transversely and develops a short, but tall, external occipital crest. Halfway towards the foramen magnum, the supraoccipital remains concave along the midline, but turns convex along the nuchal crest. Yet further posteriorly, the surface of the shield becomes flattened dorsal to the foramen magnum, but strongly concave transversely as it merges with the exoccipital. The exoccipital is slightly thickened, but does not extend posteriorly beyond the level of the occipital condyle. The condyles themselves are situated on a short, barely constricted neck.

**Figure 7.**
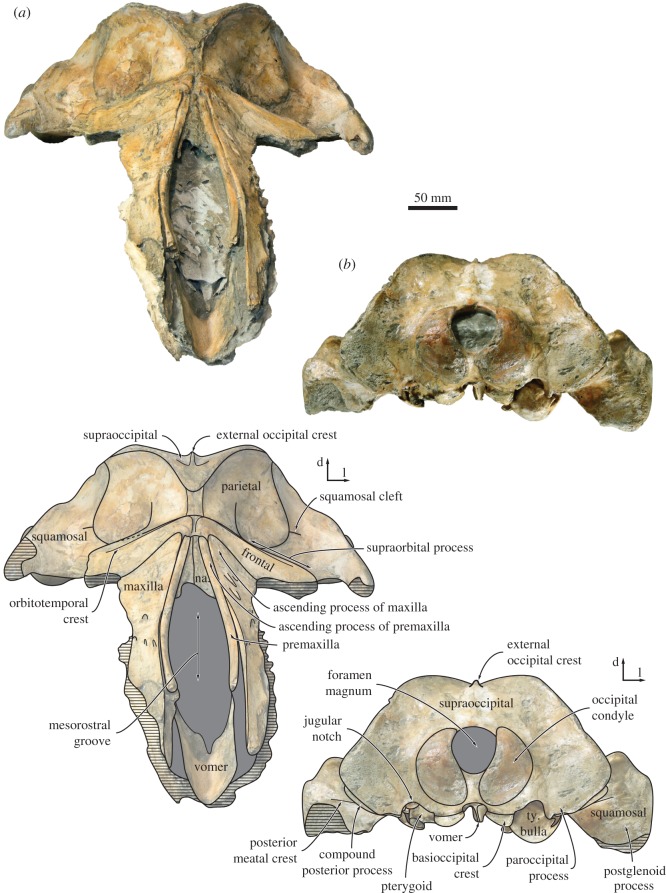
Cranium of *Tiucetus rosae* (MNHN.F. PPI261, holotype) in (*a*) anterior and (*b*) posterior view (d, dorsal; l, lateral; na., nasal; ty. bulla, tympanic bulla).

In posterior view, the foramen magnum occupies approximately two-thirds of the total height of the occipital condyles (figure 7*b*). The dorsal condyloid fossae are present, but shallow. Lateral to the condyle, the jugular notch is relatively wide and open. The paroccipital process is squared and rather narrow—considerably more so than in *Isanacetus* (MFM 28501), *Parietobalaena* (e.g. *P. palmeri*, USNM 16119; *P. yamaokai*, HMN F0042) and other cetotheriids (e.g. *Piscobalaena nana*, MNHN.F. SAS1616). The basioccipital crest is inflated and has a sharp, ridge-like medial border that partially floors the area between the choanae and the intercondyloid notch.

**Figure 8.**
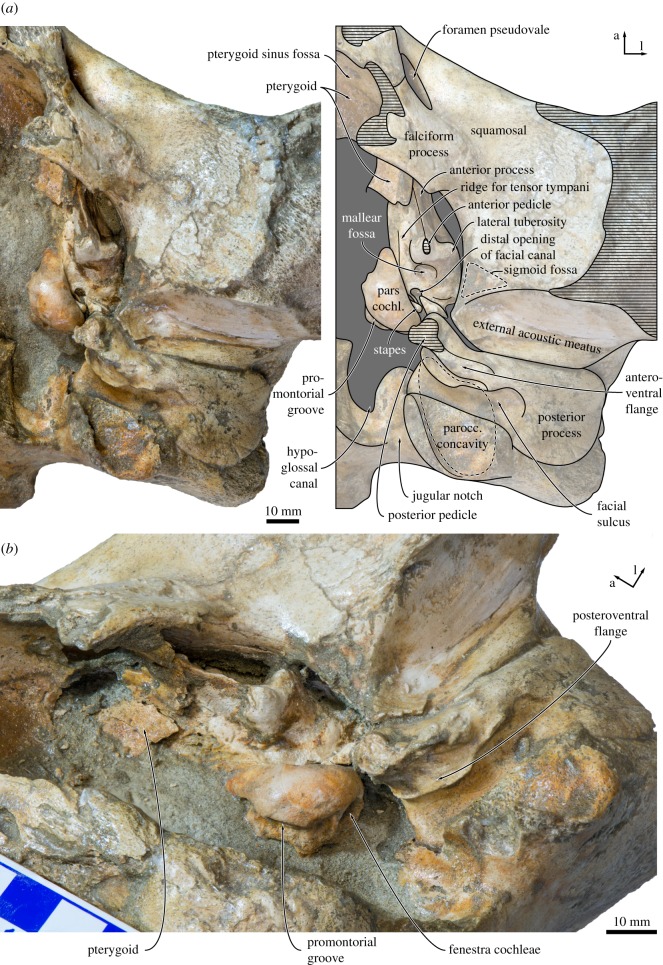
Left auditory region of *Tiucetus rosae* (MNHN.F. PPI261, holotype) in (*a*) ventral and (*b*) oblique ventromedial view (a, anterior; l, lateral; parocc. concavity, paroccipital concavity; pars cochl., pars cochlearis).

**Figure 9.**
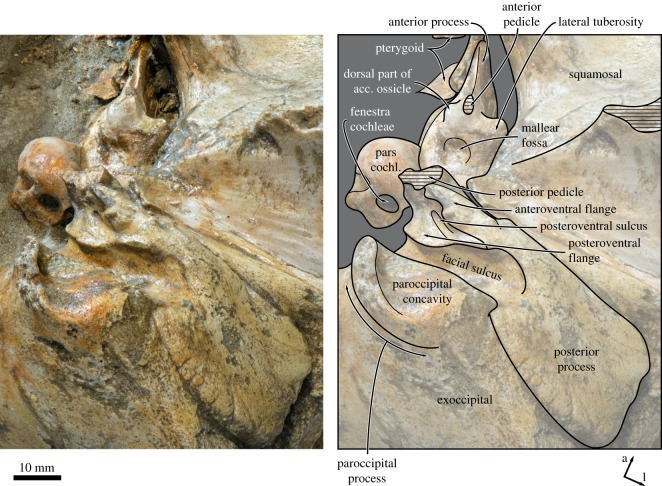
Left periotic of *Tiucetus rosae* (MNHN.F. PPI261, holotype), in posteroventral view (a, anterior; acc. ossicle, accessory ossicle; l, lateral; pars cochl., pars cochlearis).

In ventral view, the lateral edges of the basioccipital crests are approximately parallel (figure 6). The ventral condyloid fossae are virtually indistinguishable. Posterolateral to the basioccipital crest, there is a fused but still distinct medial exoccipital crest forming the medial border of the jugular notch. The jugular notch itself is rounded and, on the right side, houses a distinct hypoglossal foramen. The right hypoglossal canal is rather short, with its internal opening and the hypoglossal foramen being a mere 6 mm apart. On the left side, the hypoglossal canal appears to be anteriorly open, so that only a sulcus with no distinct dorsal or ventral foramina remains (figure 8). The ventral surface of the paroccipital process is deeply excavated by the paroccipital concavity (figures 8 and 9). As in other cetotheriids, such as *Metopocetus hunteri* (NMR9991-07729) and *Piscobalaena nana* (MNHN.F. SAS 1616), the medial border of the paroccipital concavity is defined by a tall, rounded crest, whereas the lateral side of the concavity is open. Anteriorly, the paroccipital concavity extends on to the medial portion of the posterior process, within the area defined by the anteroventral and posteroventral flanges of the periotic (see below) (figure 8).

### 4.7. Vomer

In dorsal view, the trough-like lateral sheets of the vomer floor the mesorostral groove (figure 2). In ventral view, the portion of the vomer between the maxillae is transversely rounded and smooth (figure 6). As preserved, the vomer is clearly exposed along the midline of the rostrum, but obvious lateral displacement of the maxillae makes it unclear whether this was also the case in life. Immediately posterior to the maxillo-palatine suture, the vomer narrows and develops a faint, obliquely oriented lateral ridge on each side. The latter extends from the maxilla almost to the level of the choanae, and marks the outline of the vomero-palatine suture. Somewhat medial to the ridge, two well-defined, anteriorly directed sulci probably represent the palatine canal (figure 6).

Dorsal and posterior to the vomero-palatine suture, the vomer is laterally excavated by the narial passages. Ventrally, the part of the vomer covered by the palatines bears a sharp vomerine crest (figure 6). At the level of the choanae, this crest expands into an elongate, lozenge-shaped platform, but then abruptly narrows again and rises towards the level of basioccipital. The posteriormost portion of the vomer is extremely narrow and tightly wedged between the medial laminae of the pterygoids. The posterior edge is broken, but appears to have been aligned with the anterior quarter of the basioccipital crests. As far as can be told, the vomerine crest extends posteriorly to the end of the vomer, or very nearly so, remaining sharp along the entire way.

### 4.8. Pterygoid

The right pterygoid is nearly complete, except for the hamular process and some minor damage to the rim of the Eustachian notch (figure 6). In ventral view, the pterygoid is broadly exposed between the palatine and the falciform process of the squamosal, and forms approximately one-quarter of the rim of the foramen pseudovale (figure 8*a*). Laterally, a small portion of the pterygoid extends on the wall of the temporal fossa (figure 5). The base of the hamular process is thin dorsoventrally and located directly beneath the Eustachian notch. The medial lamina of the pterygoid is relatively broad, with its ventral surface being slightly concave transversely. The pterygoid sinus fossa is relatively narrow, extends anteriorly to approximately the same level as the foramen pseudovale, and is entirely roofed by the dorsal and lateral laminae (figure 6). Posteriorly, the lateral lamina extends on to the anterior process of the periotic and covers at least half of its medial surface (figure 8).

### 4.9. Alisphenoid

In lateral view, the area of the temporal fossa exposing the alisphenoid is damaged. Nevertheless, it appears that the external surface of this bone occupies a relatively small space between the parietal, squamosal and pterygoid, and—as far as can be told—anteriorly contributes to the rim of the orbital fissure. Details of this region, including the position and size of the orbitosphenoid, are too poorly preserved for further description.

### 4.10. Periotic

Both periotics are preserved *in situ*, but only the ventral surface of the right periotic is currently accessible. In ventral view, the anterior process is slightly longer than the pars cochlearis and blade-like, with its entire ventral border forming a sharp crest (figures 8 and 9). A laterally compressed anterior process also occurs in some cetotheriids, such as *Herpetocetus* (e.g. *H. transatlanticus*, USNM 182962) and *Kurdalagonus mchedlidzei* (NMRA 10476/1), but strongly contrasts with the markedly inflated posterior portion of the anterior process in *Parietobalaena* (e.g. *P. palmeri*, USNM 10668, 16119). There is no anterior bullar facet. The anterior pedicle is aligned with the anterior border of the pars cochlearis, and firmly fused to the body of the periotic. A rounded tubercle immediately above the pedicle (figure 9) is presumably homologous with the fused dorsal portion of the accessory ossicle, but there is no evidence of a suture or any part of the fovea epitubaria. The lateral tuberosity is robust, short, somewhat pointed and located posterolateral to the anterior pedicle. The border of the mallear fossa is indistinct. The distal opening of the facial canal is located slightly anterior to the fenestra vestibuli. Both the latter and the fossa for the stapedial muscle are obscured by the *in situ* stapes.

The pars cochlearis is bulbous, with a smooth ventral surface and a rounded anteromedial corner (figure 8). The fenestra cochleae is flush with the medial and posterior borders of the pars cochlearis, rather than being recessed as in most balaenopterids. The posterior process is elongate, somewhat squared in outline and slightly expanded distally. The facial sulcus occupies the posterior half of the posterior process, and is partially floored by a narrow posteroventral flange (*sensu* [7]) (figure 9). Along its anterior border, the posterior process furthermore bears a well-developed anteroventral flange (*sensu* [7]), which delimits the extension of the paroccipital concavity anterior to the facial sulcus, and may correlate with the presence of an enlarged cartilaginous tympanohyal [15]. A similar condition exists in virtually all known cetotheriids [15], but is absent or rudimentary in *Parietobalaena* (e.g. *P. palmeri*, USNM 10668, 16119; *Parietobalaena* sp. SMNH VeF62). Laterally, the anteroventral flange becomes lower and terminates approximately 13 mm before the end of the facial sulcus.

In medial view, the outline of the anterior process is obscured by matrix and the lateral lamina of the pterygoid, but it appears that at least the anteroventral angle is pointed and triangular (figure 8*b*). Anterior to the pars cochlearis, the anterior process bears an approximately 12 mm long ridge for the origin of the tensor tympani muscle. A deep, step-like promontorial groove runs across the entire length of the pars cochlearis and terminates just anterior to the fenestra cochleae in a large (approx. 5 mm), posteromedial embayment. There is no posterior cochlear crest, and the pars cochlearis consequently does not approach the crista parotica. In posterior or posterolateral view, the fenestra cochleae is circular and well separated from the aperture for the cochlear aqueduct (figure 9).

On the posterior process, just posterior to the anteroventral flange, a small (1 mm) foramen opens into a *posteroventral sulcus* (new term) that runs across the ventral surface of the posteroventral flange (figure 9). Lateral to the facial sulcus, the outer portion of the posterior process slightly widens, abruptly flattens and becomes oriented dorsolaterally, thus forming a distinct external surface that is exposed on the lateral skull wall. The same condition, often more pronounced, is characteristic of all cetotheriids [1,3]. A somewhat similar morphology also occurs in some specimens of *Parietobalaena* (e.g. *P. palmeri*, USNM 10668), but the external surface of the posterior process, if present at all, is generally smaller, less well defined, not laterally expanded and not clearly offset from the facial sulcus.

### 4.11. Tympanic bulla

**Figure 10.**
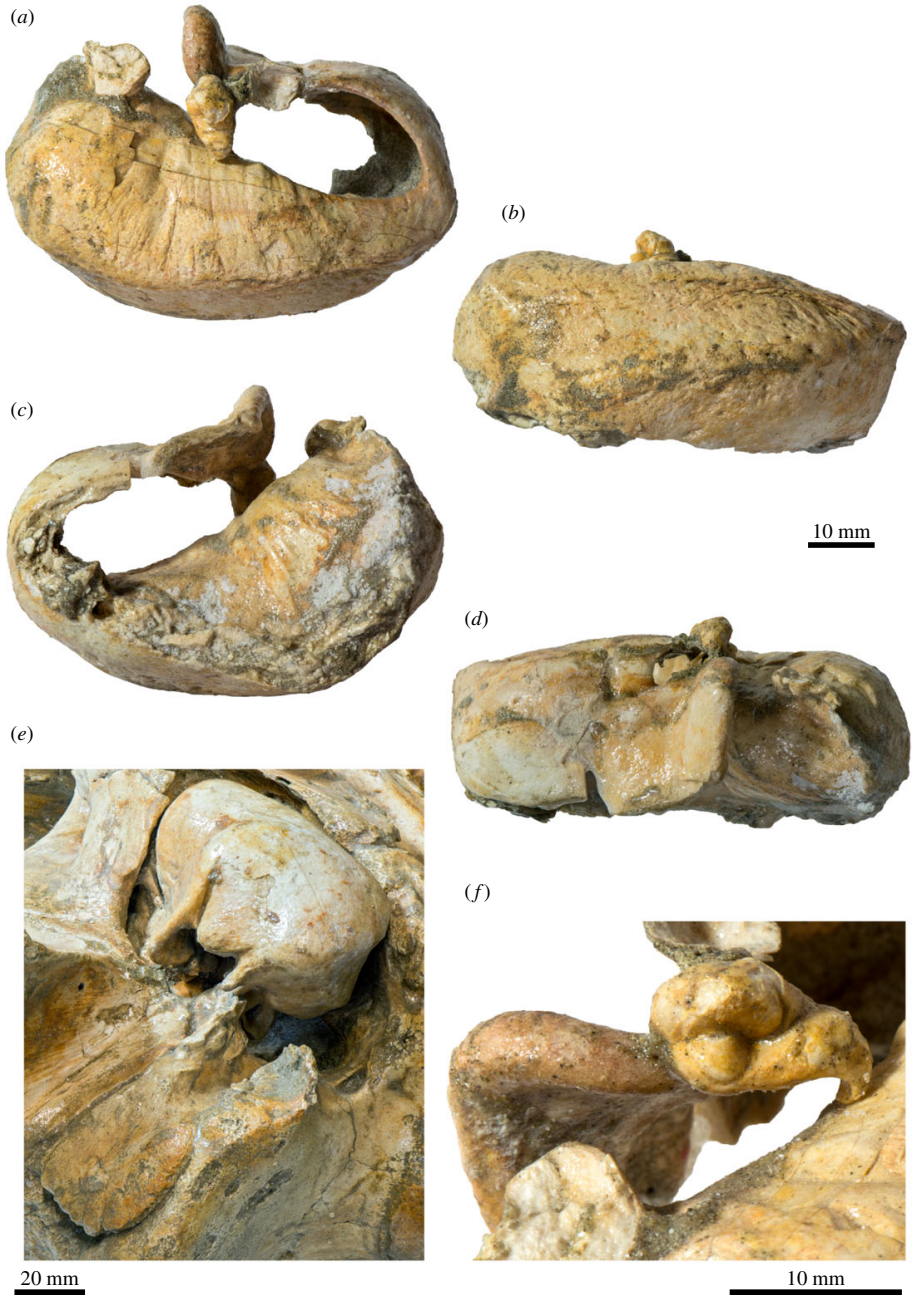

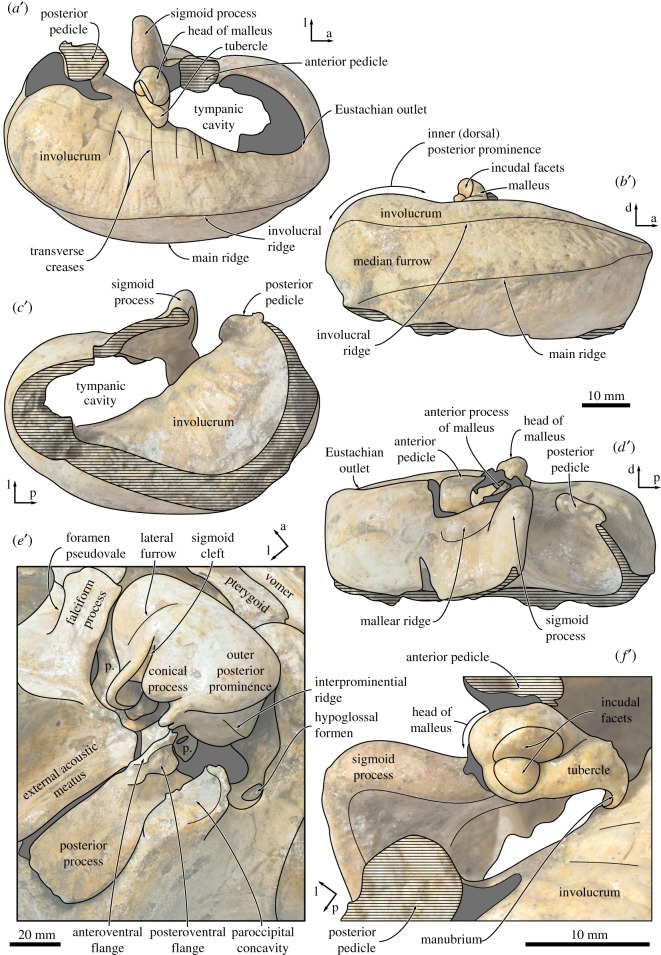
Tympanic bulla and malleus of *Tiucetus rosae* (MNHN.F. PPI261, holotype). Left tympanic bulla in (*a*) dorsal, (*b*) medial, (*c*) ventral (revealing the inside of the tympanic cavity) and (*d*) lateral view; (*e*) right tympanic bulla in oblique posterolateral view; (*f*) left malleus in posterior view (p., periotic.; a, anterior; d, dorsal; l, lateral; p, posterior).

The right tympanic bulla is complete and preserved *in situ*, whereas the left bulla is detached but missing its ventral half and the posterior portion of the outer lip. In dorsal view (figure 10*a*), the involucrum is narrow adjacent to the Eustachian outlet and then gradually widens posteriorly. There is no sign of an involucral incisure (*sensu* [33]), but the involucrum does bear fine transverse sulci all along its dorsal surface. The involucral and main ridges (*sensu* [14]) are convex medially.

The portion of the outer lip anterior to the lateral furrow is inflated and rounded (figure 10*e*). The dorsal border of the sigmoid process is oriented approximately transversely, not obviously twisted, and located at just under two-thirds of the total length of the bulla. Posteriorly, the sigmoid process does not overhang the conical process, but is connected to it via a narrow ridge. As far as can be told, the conical process itself is straight and narrow, with its apex being neither thickened nor deflected. The posterior pedicle is elongate and located close to the posterior border of the bulla, with no sign of a posterior extension of the involucrum as seen in, for example, *Balaenoptera musculus* [34]. Internally, the pedicle is excavated by a branch of the tympanic cavity that probably marks the ancestral position of the posterior sinus [15].

In lateral view, the lateral furrow is well developed, step-like and oriented approximately vertically (figure 10*d*). Dorsal to the furrow, the mallear ridge is low and narrow, and oriented posterodorsally. The sigmoid cleft is straight and also largely vertical, resulting in the absence of a distinct ventral border of the sigmoid process (figure 10*e*). The conical process is parabolic in outline, and separated from the posterior pedicle by a narrow, posterolaterally directed channel.

In medial view, the posterior portion of the bulla is divided into inner and outer posterior prominences (figure 10*b*). As in all described chaeomysticetes except eomysticetids, the bulla has rotated medially around its long axis (relative to the condition in archaeocetes), so that the inner posterior prominence now faces dorsally, and the outer posterior prominence ventrally. As a result, the dorsal surface of the involucrum is divided into (i) a slightly depressed anterior portion marking the position of the Eustachian outlet; (ii) a horizontal central portion; and (iii) the bulbous inner (dorsal) posterior prominence. The involucral and main ridges are well developed and separated from each other by a broad, but relatively shallow, median furrow. The main ridge is oriented obliquely and traverses the medial face of the bulla from its posteroventral to its anterodorsal corner. At the level of the Eustachian outlet, the main and involucral ridges converge.

In ventral view, the anteromedial corner of the bulla is somewhat angled and the ventral surface of the bulla transversely convex (figures 6 and 10*c,e*). There is no anterolateral shelf. Inside the tympanic cavity, the posterior portion of the involucrum bears a series of posteromedially oriented ridges. Unlike in toothed mysticetes and archaic chaeomysticetes, there is no internal transverse ridge arising from the ventral portion of the involucrum (figure 10*c*). The tympanic sulcus is obscured on the right and mostly lost on the left, but its beginnings can be traced on the posterior surface of the sigmoid process. Judging from this limited evidence, the tympanic sulcus appears to descend more steeply on its way towards the conical process than in other cetotheriids, such as *Piscobalaena nana* (MNHN.F. PPI259, SAS892). In posterior view, the median furrow terminates at an obliquely oriented interprominential ridge (figure 10*e*). There is no elliptical foramen.

### 4.12. Malleus and stapes

In anterior view, the anterior process of the malleus is robust and excavated by the groove for the chorda tympani. Dorsally, the anterior process is closely apposed to the dorsomedial corner of the sigmoid process, without, however, being entirely fused to it as in recent balaenopterids. At its base, the anterior process is surrounded by a weakly developed rim, which posteriorly appears to be confluent with the tympanic sulcus. In posterior view, the head of the malleus is rounded and separated from the tubercle by a deep oblique groove (figure 10*f*). The incudal facets are perpendicular to each other, with the vertical facet being roughly twice as large as the horizontal one. The vertical facet is flattened, whereas its horizontal counterpart is convex and somewhat dome-like. The tubercle is oriented medially and terminates in a ventrally curved, pronounced manubrium. The muscular process appears to be indistinct. Most of the stapes is obscured by its *in situ* position inside the fenestra vestibuli. Nevertheless, as far as can be told, the head of the stapes is separated from the crura by a short neck, and the stapedial foramen is patent.

## 5. Discussion

### 5.1. Phylogeny

Mysticete taxonomy has traditionally been plagued by the overuse of Cetotheriidae as a wastebasket taxon covering all fossil mysticetes outside the extant families. Over the past decade, a thorough re-examination addressed this problem by redefining Cetotheriidae as a clade of mostly Late Miocene–Pliocene species related to *Cetotherium rathkii* [1–3]. Nevertheless, the leftovers of this revision – the ‘cetotheres’ *sensu lato* – have remained an evolutionary conundrum. ‘Cetotheres’ *sensu lato* include taxa such as *Aglaocetus*, *Cophocetus*, *Diorocetus*, *Isanacetus*, *Parietobalaena*, *Pelocetus, Thinocetus* and *Uranocetus*, none of which appear to share unequivocal similarities with either each other or any of the established families. This lack of obvious affinities is reflected across numerous phylogenetic studies, which have placed ‘cetotheres’ *sensu lato* either inside [2,4,6,11,35,36] or outside [1,9,37] crown Mysticeti, as sister to [9,11,35] or apart [1,2,4,36] from actual cetotheriids, and within either a single clade [11,35,36] or not [1,2,4,6,9,37].

**Figure 11.**
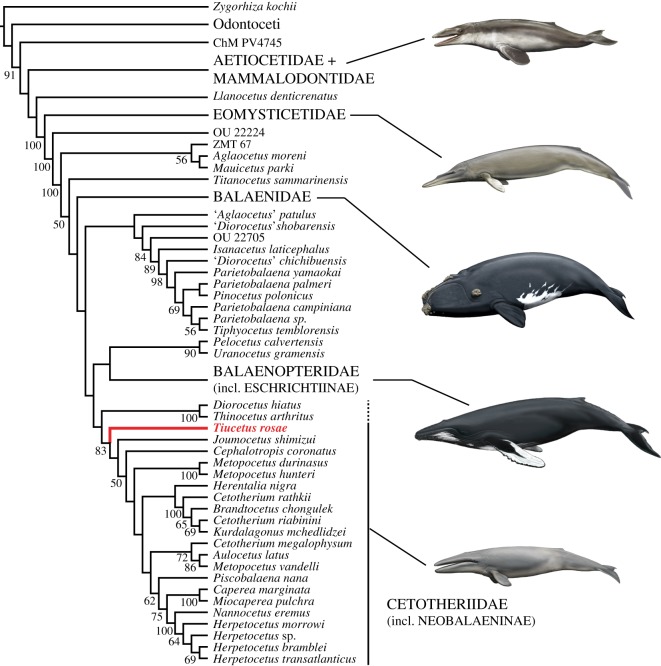
Phylogenetic position of *Tiucetus rosae* (shown in red) among other living and extinct baleen whales.

Most studies agree that some or all crown mysticete lineages originate from within ‘cetotheres’[6,8,24], but the structure of this early phase of baleen whale evolution has remained largely obscure. Up to a point, this situation is unavoidable: basal taxa share fewer of the defining features of the clade they belong to, and hence are also more difficult to identify. In this context, the particular morphology of *Tiucetus rosae* is both striking and informative. Our phylogenetic analysis recovers *Tiucetus* as the basalmost cetotheriid (figure 11), in line with its relatively large posterior process, enlarged paroccipital concavity, and possession of a posteroventral sulcus (see below). At the same time, the morphology of the cranial vertex of *Tiucetus* entirely differs from that of typical cetotheriids—e.g. in lacking posteriorly convergent ascending processes of the maxillae, and in having a large exposure of the parietal on the vertex—and instead closely resembles that of *Diorocetus* and *Parietobalaena*. *Tiucetus* therefore*,* more than any other described species, bridges the morphological gap between ‘cetotheres’ *sensu lato* and one of the major mysticete families, and sheds light on the cranial architecture of basal cetotheriids.

Sister to, and possibly included within, Cetotheriidae is a previously identified clade comprising *Diorocetus hiatus* and *Thinocetus arthritus* [2,4]. Both species resemble cetotheriids in having a relatively large posterior process, a deep facial sulcus, and a somewhat posteriorly elongated angular process of the mandible bearing a fossa for the medial pterygoid muscle (as inferred for *Herpetocetus morrowi* [6]). Moreover, the placement of *D. hiatus* as a basal cetotheriid is supported by the results of another recent analysis [8]. Nevertheless, the phylogenetic position of *D. hiatus* has generally been extremely variable, leading to its interpretation as a stem balaenopteroid [2,10,15], stem plicogulan [38], sister to a clade comprising balaenopteroids and cetotheriids [6,39], or member of a clade including both cetotheriids and other ‘cetotheres’ *sensu lato* [9,11]. Given this level of disagreement, we prefer not to formally reassign *Diorocetus* and *Thinocetus* to Cetotheriidae, pending a more detailed re-examination of these species.

More broadly, our results group cetotheriids with balaenopteroids, to the exclusion of a clade comprising *Isanacetus*, *Parietobalaena* and related taxa. Several members of the latter share distinctive traits such as (i) a sharply pointed mandibular foramen (in ‘*Diorocetus’ chichibuensis, Parietobalaena palmeri*, *P. yamaokai* and *Parietobalaena* sp. SMNH VeF62; unclear in *P. campiniana* owing to breakage); (ii) a hypertrophied body of the periotic (in ‘*Aglaocetus*’ *patulus*, ‘*D.*’ *chichibuensis, Isanacetus laticephalus, P. campiniana, P. palmeri* and *P. yamaokai*); and (iii) a tympanic bulla with an anteriorly retracted involucral ridge (in *P. palmeri*, *P. yamaokai*, *Pinocetus polonicus* and, possibly, *Tiphyocetus temblorensis;* less distinct in *I. laticephalus*; unclear in *P. campiniana*). Nevertheless, most of these species remain poorly known, owing to various issues with the known (type) material: the holotypes of ‘*Aglaocetus*’ *patulus*, ‘*Diorocetus*’ *shobarensis*, *Parietobalaena yamaokai* and *Tiphyocetus temblorensis* are badly damaged; those of *Parietobalaena campiniana* and *P. palmeri* are juveniles; and the periotic morphology remains largely or entirely unknown for ‘*Diorocetus*’ *chichibuensis*, ‘*D.*’ *shobarensis*, *Pinocetus polonicus* and *T. temblorensis*. Further preparation and redescription, e.g. of one of the paratypes of *P. yamaokai* (HMN F00042), is required to clarify taxonomy and relationships.

### 5.2. Posteroventral sulcus

**Figure 12.**
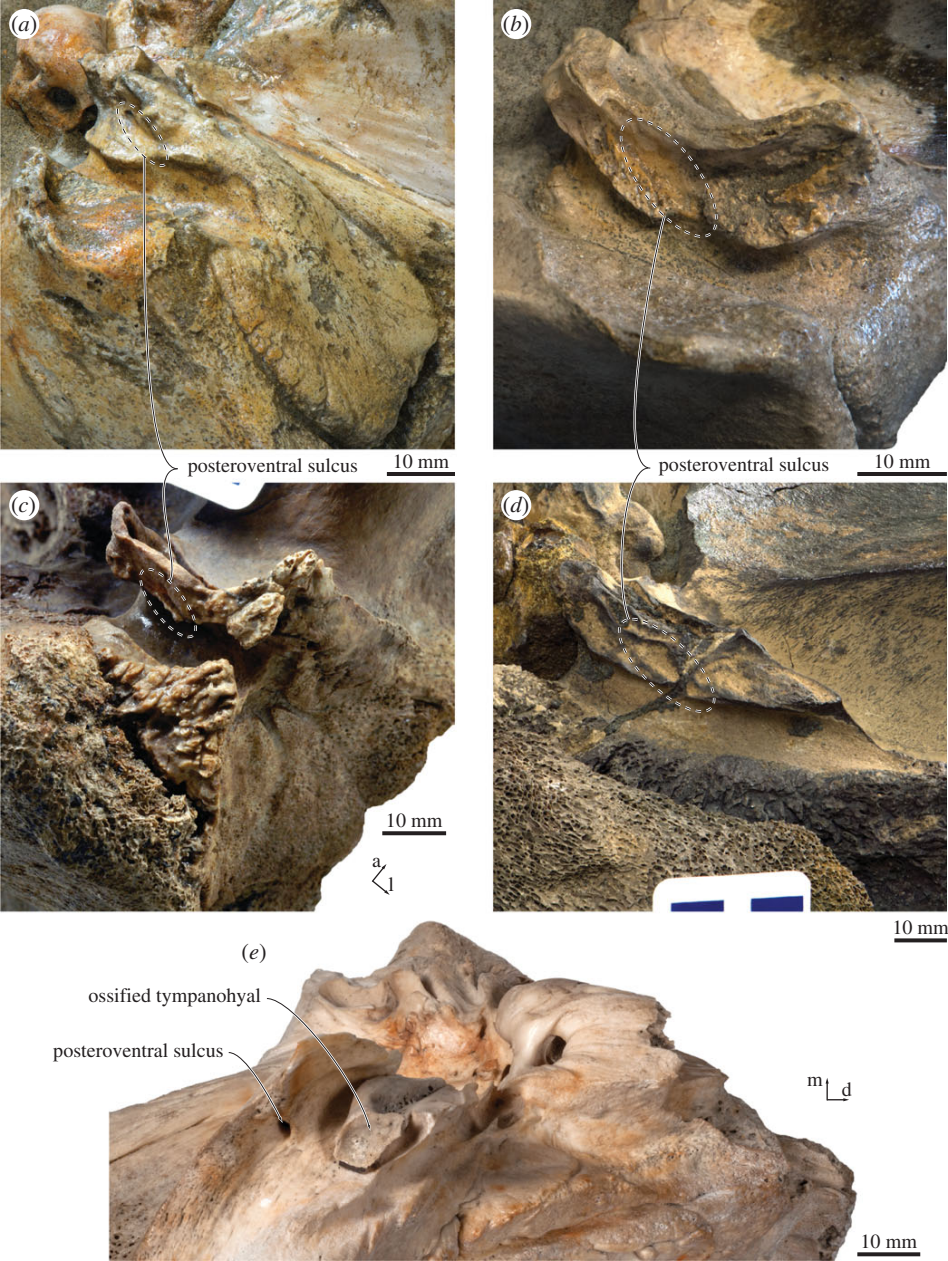
Location of the posteroventral sulcus in (*a*) *Tiucetus rosae* (MNHN.F. PPI261, holotype), (*b*) *Piscobalaena nana* (MNHN.F. SAS1616), (*c*) *Herpetocetus bramblei* (UCMP 82465), (*d*) *Herentalia nigra* (ZMA 5069, holotype) and (*e*) *Eubalaena australis* (OU 22802) (a, anterior; d, dorsal; l, lateral; m, medial).

The posteroventral sulcus on the posterior process of *Tiucetus rosae* obliquely traverses the ventral surface of the posteroventral flange, and divides the latter into an outer and an inner portion (figure 12). A similar sulcus and division occurs, seemingly without an associated foramen, in the cetotheriids *Herentalia nigra* (ZMA 5069), *Herpetocetus bramblei* (UCMP 82465) and *Piscobalaena nana* (e.g. MNHN.F. SAS1616), as well as, probably, *Herpetocetus transatlanticus* (USNM 182962) and *Metopocetus durinasus* (USNM 8518) (figure 12); and with an associated foramen in various balaenopterids (e.g. *Balaenoptera bonaerensis*, OM VT3057; *B. physalus*, OMNH, no number; *Megaptera novaeangliae*, NMNZ MM000228), *Eubalaena australis* (OU 22802) and, probably, an as yet undescribed specimen close to *Herentalia* (MAB 1323).

In balaenopterids, the sulcus and associated foramen are often small and easily missed. It thus seems plausible that these features may have been overlooked in a range of other taxa, especially in fossil specimens where breakage or even a thin layer of matrix could easily obscure their presence. In cetotheriids, the sulcus appears particularly broad and well-defined, and in this form may characterize either the family, or a subclade within it. The function of the foramen and sulcus currently remain uncertain, although their location is suggestive: in *Eubalaena*, the sulcus opens near the unusually large, ossified tympanohyal (figure 12*e*), which is rivalled in size only by *Metopocetus* [7]; in balaenopterids and cetotheriids, it opens into the anterior portion of the paroccipital concavity, which likewise may house an enlarged cartilaginous tympanohyal [15]. Together, these observations suggest that the posteroventral foramen and sulcus may carry nerves or blood vessels associated with the articulation of the hyoid apparatus to the basicranium. MicroCT scans of isolated periotics and dissections of the auditory region of extant mysticetes may shed further light on this issue.

## 6. Conclusion

*Tiucetus rosae* is a new species of extinct baleen whale that bridges the morphological gap between Cetotheriidae and the poorly understood group of Miocene chaeomysticetes often referred to as ‘cetotheres’ *sensu lato*. *Tiucetus* is the first mysticete to be described from the basal portion of the Pisco Formation (P0 *sensu* [23]), and provides insights into a fossil assemblage with a much more archaic aspect than hitherto known from this unit. The presence of a posteroventral sulcus on the posterior process represents a previously unrecognized, taxonomically widespread feature that may be associated with the articulation of the hyoid apparatus to the basicranium. The sulcus is particularly well developed in cetotheriids, and may thus help to characterize either the family or a part thereof.

## Data accessibility

The full matrix including the codings for our new material is available from MorphoBank, project 2648 (full matrix stored in the ‘Documents’ section). This published work and the nomenclatural acts it contains have been registered in ZooBank. The LSID for this publication is: urn:lsid:zoobank.org:pub:CD23FB7B-C0FD-4A6B-98D3-E723E18E8970.
